# The Role of PPARs in Lung Fibrosis

**DOI:** 10.1155/2007/71323

**Published:** 2007-07-04

**Authors:** Heather F. Lakatos, Thomas H. Thatcher, R. Matthew Kottmann, Tatiana M. Garcia, Richard P. Phipps, Patricia J. Sime

**Affiliations:** ^1^Department of Environmental Medicine, University of Rochester, Rochester, NY 14642, USA; ^2^Lung Biology and Disease Program, University of Rochester, Rochester, NY 14642, USA; ^3^Department of Medicine, University of Rochester, Rochester, NY 14642, USA; ^4^Department of Microbiology and Immunology, University of Rochester, Rochester, NY 14642, USA

## Abstract

Pulmonary fibrosis is a group of disorders characterized by accumulation of scar tissue in the lung interstitium, resulting in loss of alveolar function, destruction of normal lung architecture, and respiratory distress. Some types of fibrosis respond to corticosteroids, but for many there are no effective treatments. Prognosis varies but can be poor. For example, patients with idiopathic pulmonary fibrosis (IPF) have a median survival of only 2.9 years. Prognosis may be better in patients with some other types of pulmonary fibrosis, and there is variability in survival even among individuals with biopsy-proven IPF. Evidence is accumulating that the peroxisome proliferator-activated receptors (PPARs) play important roles in regulating processes related to fibrogenesis, including cellular differentiation, inflammation, and wound healing. PPAR*α* agonists, including the hypolidipemic fibrate drugs, inhibit the production of collagen by hepatic stellate cells and inhibit liver, kidney, and cardiac fibrosis in animal models. In the mouse model of lung fibrosis induced by bleomycin, a PPAR*α* agonist significantly inhibited the fibrotic response, while PPAR*α* knockout mice developed more serious fibrosis. PPAR*β*/*δ* appears to play a critical role in regulating the transition from inflammation to wound healing. PPAR*β*/*δ* agonists inhibit lung fibroblast proliferation and enhance the antifibrotic properties of PPAR*γ* agonists. PPAR*γ* ligands oppose the profibrotic effect of TGF-*β*, which induces differentiation of fibroblasts to myofibroblasts, a critical effector cell in fibrosis. PPAR*γ* ligands, including the thiazolidinedione class of antidiabetic drugs, effectively inhibit lung fibrosis in vitro and in animal models. The clinical availability of potent and selective PPAR*α* and PPAR*γ* agonists should facilitate rapid development of successful treatment strategies based on current and ongoing research.

## 1. INTRODUCTION

Pulmonary fibrosis is a potentially fatal disease characterized by accumulation of scar tissue in the lung interstitium, resulting in loss of alveolar function, destruction of normal lung architecture, and respiratory distress [1–3]. Known causes include inhalation of dusts and other particulates such as silica and asbestos, chemo- and radiation therapy, autoimmunity, hypersensitivity pneumonitis, and sarcoidosis [4, 5]. The idiopathic interstitial pneumonias, as the name suggests, are a group of fibrotic diseases of unknown etiology, the commonest of which is the usual intersitial pneumonitis (UIP), also called idiopathic pulmonary fibrosis (IPF) [6–8]. Some types of fibrosis respond to corticosteroids but many are refractory [9–11]. Prognosis is varied, but can be poor. UIP is considered to be the most severe of the idiopathic interstitial pneumonias. However, there is significant variability in the natural history of this disease. For example, the mean survival time after a diagnosis of UIP is less than three years [12], but there are patients who can survive for much longer periods of time with much slower (or rarely no) progression of their lung disease [13]. In contrast, other patients can develop acute exacerbations of their pulmonary fibrosis with the rapid onset of dyspnea, new radiographic abnormalities, respiratory failure, and death in 20%–86% of patients. Histological examination of their lungs reveals diffuse alveolar damage superimposed on a background of UIP [12]. The etiology of these exacerbations is unclear, but factors including infection have been implicated.

At the cellular level, pulmonary fibrosis is characterized by proliferation and accumulation of fibroblasts and scar-forming myofibroblasts in the lung interstitium with increased synthesis and deposition of extracellular matrix proteins including collagen and fibronectin [9, 14]. Although fibroblasts were previously regarded as simple structural cells, they are now recognized as having important sentinel and regulatory functions and are a rich source of regulatory cytokines and chemokines [15]. Fibroblasts differentiate to myofibroblasts after appropriate stimuli, including transforming growth factor (TGF)-*β*1 [9, 14, 16]. Myofibroblasts have some of the characteristics of smooth muscle cells, including contractility and expression of *α*-smooth muscle actin (*α*-SMA) [14, 17, 18]. The differentiation of fibroblasts to myofibroblasts, along with increased cellular proliferation and matrix deposition, leads to the development of fibroblastic foci similar in appearance to the early stages of normal wound healing. Fibrosis is usually progressive, leading to destruction of the normal lung architecture [2, 14, 17, 18]. Other organs can develop fibrosis, including the skin, liver, kidney, and pancreas, and the cellular events and signals are likely to be similar.

It has been hypothesized that fibrosis is a consequence of abnormal regulation of wound repair [2, 19, 20]. An injury leads to acute inflammation, followed by an initial repair phase in which fibroblasts and myofibroblasts at the injury site replace damaged tissue with scar tissue. Normally, this phase of wound repair is self-limiting, with myofibroblasts eventually undergoing apoptosis, and the scar tissue may be remodeled and reconstructed as relatively normal functional tissue. In fibrosis, the fibroblasts and myofibroblasts do not undergo apoptosis, but continue to proliferate, resulting in progressive scarring. The cellular signals involved in the maintenance of the profibrotic phenotype are unknown, although it is likely that TGF-*β* is a critical factor [21–24].

## 2. PPARs AND LUNG DISEASE

Peroxisome proliferator-activated receptors (PPARs) are ligand-activated transcription factors belonging to the nuclear hormone receptor family, that function to regulate a wide range of physiological activities [25]. Three different isoforms of PPARs have been identified: PPAR*α* (NR1C1), PPAR*β*/*δ* (NUC1; NR1C2), and PPAR*γ* (NR1C3), encoded by three separate genes. The PPARs and their obligate coreceptors, the retinoid X receptors (RXRs), bind a variety of ligands. The ligand-activated heterodimeric complexes then induce expression of target genes carrying peroxisome proliferators response elements (PPREs) in their promoters. PPAR*α* was first identified as the mediator of the response to peroxisome proliferators in rodents [26]. Over the past decade, PPARs have been implicated as important regulators of various physiological processes, such as lipid and lipoprotein metabolism, glucose homeostasis, cellular proliferation, differentiation, and apoptosis. PPAR*α* is found in high levels in liver, kidney, heart, and muscle, whereas PPAR*β*/*δ* is ubiquitously expressed [26, 27]. PPAR*γ* is found in two main isoforms, PPAR*γ*1 and PPAR*γ*2, derived from different pre-mRNA splice variants that use different transcription start sites. PPAR*γ* is widely expressed, and has been found in blood cells, such as macrophages [28], T and B lymphocytes [29, 30] and platelets [31], as well as in tissues including adipose, colon, spleen, retina, skeletal muscle, liver, bone marrow, and lung [27]. Within the lung, PPAR*γ* is expressed by the epithelium, smooth muscle cells, fibroblasts, endothelium, macrophages, eosinophils, and dendritic cells [32].

The role of the PPARs in lung disease is not yet clear. Both PPAR*α* and PPAR*γ* have been localized in lung tissue, including bronchial epithelial cells, alveolar walls, and alveolar macrophages [27, 32, 33]. A comparison of nonsmokers, smokers with chronic obstructive pulmonary disease (COPD), and smokers without COPD found no statistically significant difference in the number of PPAR*γ*-positive macrophages, but found an increased number of PPAR*α*-positive alveolar macrophages in smokers with COPD [34]. Sarcoidosis and pulmonary alveolar proteinosis are two other disorders in which alveolar macrophages are deficient in PPAR*γ* [35]. A causal relationship has not been determined, however, treatment of pulmonary alveolar proteinosis with granulocyte-macrophage colony-stimulating factor (GM-CSF) restores alveolar macrophage PPAR*γ* levels [36].

There is evidence that the PPARs, particularly PPAR*α* and PPAR*γ*, play a role in regulating inflammation. For example, fatty-acid-derived inflammatory mediators, including prostaglandins and leukotrienes, are ligands for PPAR*α* and *γ* [37]. Although the pathogenesis of fibrosis appears to be distinct from inflammation, and many forms of fibrosis are refractory to anti-inflammatory therapies such as corticosteroids, recent work has supported the hypothesis that fibrosis is a consequence of a dysregulated wound healing process with an initial injury and inflammatory response. Certainly, many important inflammatory signals and mediators, particularly TGF-*β*, TNF-*α*, and IL-1*β*, and prostaglandins, play key roles in fibrosis [21–24]. This review will discuss recent reports examining the link between PPARs and fibrosis, and the possibility of using PPAR ligands as antifibrotic therapies. Because the study of PPARs in lung fibrosis is relatively new, we will also review selected results from fibrotic disease models in other organs.

## 3. PPAR*α*

PPAR*α* was originally cloned as the molecular target for the hypolipidemic fibrate drugs, although arachidonic acid metabolites (eicosanoids, prostaglandins, and leukotrienes) are also important ligands [38]. PPAR*α* plays a key role in lipid metabolism and is highly expressed in tissues involved in lipid and cholesterol metabolism, including the liver, kidney, and macrophages. PPAR*α* ligands have important anti-inflammatory properties, although some studies have reported proinflammatory effects as well [37, 39]. Little is known about PPAR*α* in lung disease, although other fibrosis models implicate PPAR*α* in regulating fibrosis.

In the liver, the PPAR*α* agonists fenofibrate and WY14643 dramatically reduced fibrosis in the thioacetamide model of cirrhosis [40]. N-3 polyunsaturated fatty acid, another PPAR*α* ligand, reduced hepatic and serum TNF-*α* levels and reduced the degree of liver injury in a rat model of nonalcoholic steatohepatitis [41]. The synthetic PPAR*α* agonist WY14643 reduced the severity of steatohepatitis in C57BL/6 mice fed a methionine- and choline-deficient diet, with reductions in hepatic mRNA levels of collagen alpha 1, tissue inhibitor of metalloproteinase (TIMP)-1 and TIMP-2, and matrix metalloproteinase (MMP)-13 [42].

Fenofibrate also attenuated cardiac and vascular fibrosis in pressure-overloaded rat hearts, with reductions in collagen I and III mRNA [43], and inhibited fibrotic left ventricular remodeling in mineralcorticoid-dependent hypertension [44]. The PPAR*α* agonist gemfibrozil attenuated glomerulosclerosis and collagen deposition in diabetic ApoE-knockout mice [45].

Recent reports have found significantly reduced PPAR*α* mRNA levels in lymphocytes from cystic fibrosis patients [46], while PPAR*α* knockout mice develop more severe carageenan-induced pleural inflammation [47], suggesting a connection between diminished PPAR*α*-dependent gene activation and disease pathology.

The role of PPAR*α* in lung fibrosis was investigated in mice using the bleomycin model of lung injury and fibrosis. Intratracheal instillation of the antineoplastic agent bleomycin causes acute lung inflammation that develops into severe fibrosis, with proliferation of *α*-SMA-positive myofibroblasts, increased collagen deposition, and loss of normal alveolar architecture [48, 49]. PPAR*α*-knockout mice treated with bleomycin developed more severe inflammation and fibrosis than wild-type mice, with increased immunohistochemical detection of TNF-*α* and IL-1*β*, increased apoptosis of interstitial cells, and decreased survival [50]. Treatment of wild-type mice with the PPAR*α* agonist WY-14643 enhanced survival and reduced the severity of fibrosis, as well as reducing the detection of TNF-*α* and apoptosis by immunohistochemistry. The authors concluded that endogenous PPAR*α* ligands play an important role in limiting the fibrotic response in wild-type mice, and that treatment with PPAR*α* ligands has potential as an antifibrotic therapy.

As yet, there have been no molecular mechanisms proposed to explain these results. Since bleomycin treatment results in an acute inflammatory response that later resolves into fibrosis, it is possible that PPAR*α* agonists act to inhibit fibrosis by moderating the initial inflammatory response. This could be addressed by using a fibrogenic insult that provokes minimal inflammation, such as adenovirus-mediated overexpression of TGF-*β* [24].

Interestingly, there is some evidence that the effects of PPAR*α* agonists are not entirely dependent on PPAR*α*-dependent transcription [51]. Since the above study did not report treating PPAR*α*-knockout mice with WY-14643, the issue of the PPAR*α* dependence or independence of the effect was not addressed. It should also be noted that WY-14643 is also a weak PPAR*γ* agonist [52], and PPAR*γ* agonists may have antifibrotic activity as well (discussed below). One way to investigate the PPAR*α* dependence or independence of PPAR*α* agonists would be to study their effects in PPAR*α*-knockout fibroblasts in vitro and PPAR*α*-knockout mice in vivo. Studies using additional in vivo models of fibrosis (such as thoracic radiation or inhalation of crystalline silica) should also prove informative.

## 4. PPAR*β*/*δ*

Although little is known about PPAR*β*/*δ* in the lung, PPAR*β*/*δ* does play a critical role in wound healing in the skin. PPAR*β*/*δ* expression is upregulated following skin injury. Further, PPAR*β*/*δ*-knockout mice exhibit defective in vivo wound healing, and keratinocytes from PPAR*β*/*δ*-knockout mice show decreased adhesion and migration in vitro [53]. It has been suggested that PPAR*β*/*δ* is a critical regulator of the transition from the initial inflammatory response to the later wound healing program [54].

An intriguing recent study suggested that PPAR*β*/*δ* may be a target of prostacyclin mimetics used in treating pulmonary hypertension. Treprostinil sodium activated a PPAR*β*/*δ* reporter gene and inhibited proliferation of lung fibroblasts in vitro. The effect was not seen in lung fibroblasts from PPAR*β*/*δ*-knockout mice, demonstrating that the effect was dependent on PPAR*β*/*δ* and not on the prostacyclin receptor [55]. Finally, PPAR*β*/*δ* agonists enhance the efficacy of PPAR*γ* agonists in mediating adipocyte differentiation in vitro [56], suggesting that PPAR*β*/*δ* agonists may also potentiate the antifibrotic effects of PPAR*γ* agonists discussed below.

## 5. PPAR*γ*

PPAR*γ* is expressed in many types of lung cells including fibroblasts, ciliated airway epithelial cells and alveolar type II pneumocytes, alveolar macrophages, T lymphocytes, and airway smooth muscle cells [57]. Endogenous ligands of PPAR*γ* include 15-deoxy −Δ^12,14^-prostaglandin J_2_ (15d-PGJ_2_) [58, 59], lysophosphatidic acid [60], and nitrolinoleic acid [61]. PPAR*γ* can also be activated by synthetic ligands including the thiazolidinedione (TZD) class of clinically used insulin-sensitizing drugs [62] including rosiglitizone and pioglitizone, as well as oleanic acid derivatives known as triterpenoids [63].

The anti-inflammatory properties of PPAR*γ* ligands have been well described [37, 64]. In the lung, PPAR*γ* ligands inhibit LPS-induced neutrophilia [65, 66] and allergic airway inflammation and hyperresponsiveness in a mouse model of asthma [67, 68]. PPAR*γ* ligands also inhibit the release of proinflammatory mediators from airway epithelial cells and alveolar macrophages [69, 70]. In addition, PPAR*γ* plays an important role in regulating cellular differentiation, as PPAR*γ* ligands promote differentiation of preadipocyte fibroblasts to adipocytes [58, 59, 71].

A number of studies have investigated PPAR*γ* ligands as potential antifibrotic agents in vivo. Pioglitazone reduced carbon-tetrachloride-induced hepatic fibrosis in rats, with decreases in hydroxyl proline content, procollagen I mRNA, and *α*-SMA-positive hepatic stellate cells [72]. A similar effect was observed when fibrosis was induced by a choline-deficient diet [73, 74]. Rosiglitazone inhibits cardiac fibrosis in rats [44] and kidney fibrosis in diabetic mice and rats [45]. Intriguingly, improvements in renal function have been noted in patients with type II diabetes who are treated with TZDs [75, 76].

Only a limited amount of data is available on the effects of PPAR*γ* agonists on lung fibrosis in vivo. Ciglitazone administered by nebulization in a mouse model of asthma not only reduced lung inflammation and eosinophilia, but also reduced basement membrane thickening and collagen deposition associated with airway remodeling, as well as synthesis of the profibrotic cytokine TGF-*β* [68]. This effect was abolished by concomitant use of GW9662, an irreversible PPAR*γ* antagonist. Rosiglitazone and 15d-PGJ_2_ significantly reduced mortality, inflammation, cellular infiltrates, and histological fibrosis following intratracheal administration of bleomycin [77]. Studies of the in vivo effects of PPAR*γ* agonists have been hampered by the fact that unlike PPAR*α*, homozygous germline deletion of the PPAR*γ* gene results in embryonic lethality [78]. A conditional knockout mouse, in which exon 2 of the PPAR*γ* gene has been flanked by *loxP* sites, has been developed [78], and strategies to inducibly knock out PPAR*γ* expression in the adult mouse lung prior to fibrotic insult are being explored in a number of laboratories.

**Figure 1:**
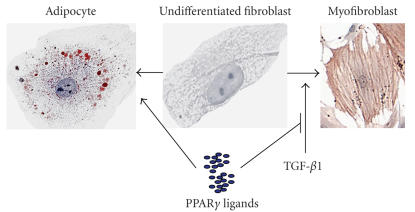
*PPAR*γ* ligands promote fibroblast differentiation to adipocytes and inhibit differentiation to myofibroblasts*. Primary human fibroblasts (center panel) can be differentiated to adipocyte-like cells (left panel) by treatment with 1 *μ*M 15d-PGJ_2_ for 8 days. Lipid droplets were visualized with oil red O staining. Alternatively, incubation with 10 ng/mL TGF-*β* for 3 days will differentiate fibroblasts to myofibroblasts (right panel). *α*-SMA was detected by immunocytochemistry. Note the long bundles of contractile fibers.

The antifibrotic effects of PPAR*γ* ligands have been studied in vitro, leading to new insights into their mechanism of action. As previously discussed, TGF-*β* drives differentiation of lung fibroblasts to myofibroblasts, a key effector cell in fibrosis [16, 23, 24]. In contrast, PPAR*γ* ligands differentiate fibroblasts to fat-storing adipocytes [58, 59]. This suggests that PPAR*γ* ligands may oppose the fibrogenic effects of TGF-*β* (Figure 1). We investigated the ability of PPAR*γ* ligands to counter the profibrotic effects of TGF-*β* on primary human lung fibroblasts. Rosiglitazone and 15d-PGJ_2_ efficiently inhibited TGF-*β*-driven differentiation of human lung fibroblasts to myofibroblasts, with reductions in the expression of *α*-SMA (a myofibroblast marker) and production of collagen [79].

Similar results have been observed in other cell types. Differentiation of hepatic stellate cells to a myofibroblast phenotype is a key step in liver fibrosis [80–82]. PPAR*γ* agonists suppress proliferation of hepatic stellate cells and chemotaxis in response to platelet-derived growth factor (PDGF) [83], and induce hepatocyte growth factor (HGF), an anti-fibrotic cytokine [84]. PPAR*γ* ligands also block PDGF-dependent proliferation, prolyl4-hydroxylase (*α*) mRNA, and the expression of collagen and *α*-SMA by pancreatic stellate cells [85]. Renal cortical fibroblasts treated with glucose induce myofibroblastic markers. Treatment of these cells with pioglitizone decreased collagen IV production, incorporation of proline, fibronectin production, and MMP-9 activity as well as reduced secretion of TIMP-1 and -2 [86, 87].

**Figure 2:**
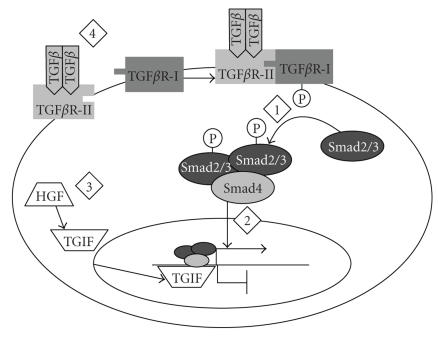
*The TGF-*β* signaling pathway*. Binding of TGF-*β* to TGF-*β* receptor II recruits TGF-*β* receptor I (TGF-*β*R-I). The kinase domain of TGF-*β*R-I phosphorylates Smad2 and 3, which form a heteromeric complex with Smad4 that translocates into the nucleus where it activates transcription of target genes. Numbers indicate points in the pathway where PPAR*γ* ligands have been demonstrated to interfere with TGF-*β* signaling. (1) GW7845, a PPAR*γ* ligand, inhibited Smad3 phosphorylation in human hepatic stellate cells [88]. (2) 15d-PGJ_2_ inhibited nuclear translocation of Smad2/3 in rat kidney fibroblasts [84]. (3) In human renal mesangial cells, 15d-PGJ_2_ induced hepatocyte growth factor (HGF), which upregulates the Smad corepressor TG-interacting factor (TGIF) [84]. (4) In mouse L929 fibroblasts, 15d-PGJ_2_ or retinoic acid upregulated the phosphatase and tensin homologue deleted on chromosome 10 (PTEN), leading to repression of TGF-*β*1 transcription [89].

The molecular mechanisms by which PPAR*γ* ligands inhibit myofibroblast differentiation and effector function are under investigation. Because TGF-*β* appears to be a key profibrotic cytokine in lung fibrosis [2, 21], several groups have investigated the ability of PPAR*γ* ligands to interfere with TGF-*β* signaling. TGF-*β* signaling is mediated by the Smad family of transcription factors [21]. Binding of TGF-*β* to type 2 TGF-*β* receptor recruits type 1 TGF-*β* receptors (TGF-*β*R-I), forming a heterotetrameric structure that phosphorylates Smad2 and Smad3. Smad2 and Smad3 form heteromeric complexes with Smad4, which translocate to the nucleus and activate transcription of target genes (Figure 2). In human hepatic stellate cells, TGF-*β* causes a time- and dose-dependent increase in Smad3 phosphorylation, followed by increased collagen production. Cotreatment with either a TGF-*β*R-I kinase inhibitor or the synthetic PPAR*γ* agonist GW7845 resulted in dose-dependent inhibition of both collagen production and Smad3 phosphorylation [88]. In contrast, the natural PPAR*γ* agonist 15d-PGJ_2_ did not inhibit nuclear translocation of Smad2/3 complexes in human renal mesangial cells treated with TGF-*β*. Instead, 15d-PGJ_2_ induced expression of the antifibrotic hepatocyte growth factor (HGF) via a peroxisome proliferator response element in the HGF promoter, and upregulated the Smad corepressor TG-interacting factor (TGIF), leading to inhibition of *α*-SMA and fibronectin expression [84]. Interestingly, the same study reported that 15d-PGJ_2_ did inhibit Smad2/3 nuclear translocation in rat kidney fibroblasts treated with TGF-*β*, while we have reported that 15d-PGJ_2_ does not inhibit TGF-*β*-stimulated phosphorylation of Smad2 in human lung fibroblasts [79]. It is possible that inhibition of myofibroblast differentiation by PPAR*γ* agonists is mediated by different mechanisms in different cell types, or that natural and synthetic agonists act by different mechanisms.

Another candidate mechanism for inhibition of profibrotic effector functions of fibroblasts involves upregulation of the tumor-suppressor phosphatase and tensin homologue deleted on chromosome 10 (PTEN). The PTEN promoter contains a PPRE, and PPAR*γ* ligands upregulate PTEN expression [90]. In vitro studies have shown that PTEN inhibits fibroblast-myofibroblast differentiation and expression of *α*-SMA and collagen in human and mouse lung fibroblasts [91], while loss of PTEN activity contributes to the migratory/invasive phenotype of lung fibroblasts isolated from IPF patients [92]. It has also been reported that PTEN levels are decreased in the lung tissue of IPF patients, and that PTEN knockout mice are more susceptible to bleomycin-induced fibrosis [91]. Interestingly, both 15d-PGJ_2_ and the RXR ligand 9-*cis*-retinoic acid inhibited transcription of the TGF-*β*1 gene via PTEN upregulation in mouse L929 fibroblasts [89], providing an additional mechanism by which PPAR*γ* ligands might interfere directly with the profibrotic effects of TGF-*β*.

One important consideration is that the effects of PPAR*γ* ligands may not all be dependent on PPAR*γ*-dependent transcriptional activation. PPAR*γ*-dependent transcriptional repression has been described in adipogensis, but not in myofibroblast differentiation [93, 94]. Additionally, recent reports have suggested that some of the biological effects of 15d-PGJ_2_ are moderated by a PPAR*γ*-independent mechanism involving modification of protein thiols by an electrophilic carbon on the imidazole ring of 15d-PGJ_2_ [95, 96]. For example, the ability of troglitazone or 15d-PGJ_2_ to inhibit proliferation of hepatic stellate cells was shown to be PPAR*γ*-independent [97], while 15d-PGJ_2_ inhibts the proliferation of human breast carcinoma cell lines by covalent modification of the estrogen receptor DNA-binding domain [98]. We examined the PPAR*γ* dependence of the antifibrotic effects of PPAR*γ* ligands on human lung fibroblasts. Neither the irreversible PPAR*γ* antagonist GW9662 nor a dominant-negative PPAR*γ* mutant significantly blocked the ability of 15d-PGJ_2_ to inhibit TGF-*β*-induced *α*-SMA expression, suggesting that this effect of 15d-PGJ_2_ was largely PPAR*γ*-independent [79]. However, the antifibrotic effects of rosiglitizone were rescued significantly by the dominant-negative PPAR*γ*, suggesting that while rosiglitizone was less effective at inhibiting myofibroblast differentiation, the effect was mostly dependent on PPAR*γ* [79].

## 6. RETINOID X RECEPTOR

The PPARs must form heterodimers with the retinoid X receptor (RXR) in order to initiate gene transcription [99]. Therefore, it has been proposed that the anti-inflammatory and antifibrotic functions of PPARs may be addressed or enhanced by RXR ligands, predominantly the retinoic acids [100, 101]. In the rat liver, endogenous and synthetic retinoic acids (RA) reduced proliferation of HSCs and production of collagen I. In addition, all-*trans* RAs inhibited the synthesis of collagen I/II and fibronectin but did not affect HSC proliferation [102]. Levels of RXR-*α* and RXR-*β* were decreased in the HSC of rats with cholestatic liver fibrosis [103]. In addition, there were decreases in all-*trans* RA and 9-*cis*-RA levels and RA binding to the retinoid receptor response element (RARE) in fibrotic liver tissue. Similar findings have been demonstrated in glomerular mesangial cells where 9-*cis*-RA induced the antifibrotic growth factor HGF and inhibited TGF-*β*-stimulated induction of *α*-SMA and fibronectin [104]. Synergistic effects between RXR ligands and PPAR ligands have not yet been reported in lung fibroblasts in vitro or in animal models of lung fibrosis, though this is under investigation.

## 7. CONCLUSION

Although the role of the PPARs in fibrosing diseases has been less well studied than their role in regulating inflammation, a number of key results have emerged. PPAR*γ* agonists inhibit the differentiation of lung fibroblasts to myofibroblasts in vitro, and also inhibit airway remodeling and fibrosis in animal models [77, 79]. PPAR*α* agonists also attenuated fibrosis in the mouse bleomycin model, while PPAR*α* knockout mice developed more severe disease [50].

Our understanding of the role of PPARs in lung fibrosis is hindered by the relative lack of experiments directly involving the lung or lung cells. However, progress has also been made toward determining the role of the PPARs in fibrosing diseases of the liver, kidney, and pancreas. Hepatic stellate cells and pancreatic stellate cells differentiate to myofibroblast-like cells under the same stimulus as lung fibroblasts, and this differentiation is inhibited by both natural and synthetic PPAR*γ* ligands [83–85]. The TZD class of PPAR*γ* agonists is effective in reducing liver, cardiac, and kidney fibrosis in rats and mice [44, 45, 72]. PPAR*α* agonists, including the fibrate drugs, have also shown promise in attenuating liver, kidney, and cardiac fibrosis [40, 43, 45].

**Figure 3:**
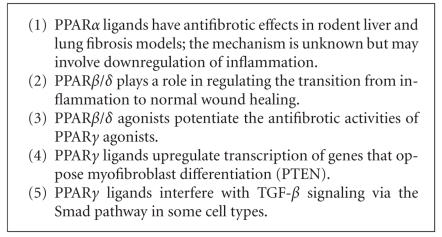
Key concepts in the regulation of fibrosis by PPARs.

The mechanisms by which PPAR ligands alter fibrosis are not well understood, but appear to involve multiple regulatory pathways (see Figure 3). Natural and synthetic PPAR*γ* agonists inhibit TGF-*β*-driven myofibroblast differentiation and activation in hepatic stellate cells, kidney fibroblasts, and lung fibroblasts. In human hepatic stellate cells, the PPAR*γ* agonist GW7845 inhibited Smad3 phosphorylation and nuclear translocation [88], while a similar result was seen with 15d-PGJ_2_ in rat kidney fibroblasts [84]. However, 15d-PGJ_2_ did not alter Smad2 phosphorylation in human lung fibroblasts [79] or human renal mesangial cells, but instead upregulated HGF and TGIF [84]. It is likely that the precise mechanism of action of PPAR*γ* ligands varies depending on the cell type and agonist used. A further complication is that PPAR*γ* agonists appear to have PPAR*γ*-independent effects. Further studies using pharmaceutical inhibitors of PPAR*γ* or PPAR*γ* knockout cell lines may prove useful in further investigations.

A very intriguing recent report found that 15d-PGJ_2_ altered transcriptional activity of the estrogen receptor by covalent modification of cysteine residues in its zinc finger DNA-binding domain [98]. Since cysteine is a ready target of covalent modification by 15d-PGJ_2_ [95, 96] and many transcription factors use cysteine-rich zinc finger DNA-binding domains, this suggests that one possible mechanism by which PPAR*γ* ligands can affect the regulation of cell differentiation independently of PPAR*γ* itself is via modification of other transcription factors.

There are less data available on the mechanism of action of PPAR*α* and *β*/*δ* agonists. Although PPAR*α* agonists attenuate animal preclinical fibrosis models, studies of the direct effect of PPAR*α* ligands on myofibroblast activation have not been reported. Treprostinil inhibition of lung fibroblast proliferation is PPAR*β*/*δ*-dependent [55], and PPAR*β*/*δ* also appears to play a role in keratinocyte maturation and function [53]. It has been hypothesized that fibrosis is a consequence of dysregulated wound healing and tissue remodeling following an initial injury [54]. This may provide the mechanistic link between PPAR*α* and *β*/*δ* and fibrosis. Rather than directly acting on fibroblasts and myofibroblasts, PPAR*α* may regulate inflammation, while PPAR*β*/*δ* regulates the transition from inflammation to wound healing [54, 105]. Thus, PPAR*α* and *β*/*δ* agonists may ameliorate fibrosis by altering the initial inflammatory response and the transition to a fibrogenic milieu, respectively.

The relationship between the PPARs and fibrosis is likely to be complex. As discussed above, PPAR*α* and PPAR*γ* are involved in regulating both inflammation and fibrosis, and some ligands have affinity for more than one PPAR. In addition, because RXR is the obligate dimerization partner for all three PPARs, modulating RXR activity may have multiple overlapping or even conflicting effects. A number of useful tools exist to study these relationships, including highly specific synthetic agonists and antagonists, dominant negative expression constructs, and germline and conditional gene knockouts. Each of these approaches has potential advantages and drawbacks. In particular, genetic ablation of PPAR genes will eliminate their function from both inflammatory and repair processes, making it difficult to determine their role in each process independently. This problem can be addressed by using multiple complimentary approaches to examine PPAR function in both normal and abnormal wound repair and fibrosis.

It must be emphasized that important classes of PPAR*α* (the fibrate drugs) and PPAR*γ* (TZDs) agonists are currently available in the clinic. Although the frequency of lung fibrosis in the general population is not high, it may be possible to perform retrospective studies of long-term users of TZDs and fibrates to determine whether these drugs reduce the incidence or severity of lung fibrosis and other fibrosing diseases. More importantly, the clinical availability of these drugs means that significant results from animal studies of fibrosis models may be rapidly applied in the clinical setting. Recent advances in drug delivery by inhalation may allow delivery of antifibrotic PPAR agonists directly to the site of fibrosis (as has already been demonstrated with the use of ciglitazone in a mouse model of airway remodeling [68]), achieving higher effective doses at the target site with lower systemic side effects. As most forms of lung fibrosis are refractory to current treatment, the rapid translation of basic research to bedside practice holds great promise for a patient population suffering from a largely untreatable disease.
